# *Notes from the Field*: Nontuberculous Mycobacteria Infections in U.S. Medical Tourists Associated with Plastic Surgery — Dominican Republic, 2017

**DOI:** 10.15585/mmwr.mm6712a5

**Published:** 2018-03-30

**Authors:** Joanna Gaines, Jose Poy, Kimberlee A. Musser, Isaac Benowitz, Vivian Leung, Barbara Carothers, Judy Kauerauf, Noreen Mollon, Monique Duwell, Kathleen Henschel, Alexandra De Jesus, Sara K. Head, Keun Lee, Nelson Arboleda, Douglas H. Esposito

**Affiliations:** ^1^Travelers’ Health Branch, Division of Global Migration and Quarantine, National Center for Emerging and Zoonotic Infectious Diseases, CDC; ^2^New York City Department of Health and Mental Hygiene, Bureau of Communicable Disease; ^3^Wadsworth Center, New York State Department of Health; ^4^Prevention and Response Branch, Division of Healthcare Quality Promotion, National Center for Emerging and Zoonotic Infectious Diseases, CDC; ^5^Epidemiology Workforce Branch, Division of Scientific Education and Professional Development, Center for Surveillance, Epidemiology, and Laboratory Services, CDC; ^6^Connecticut Department of Public Health; ^7^New Jersey Department of Health, Communicable Disease Service; ^8^Illinois Department of Public Health, Communicable Disease Control Section; ^9^Michigan Department of Health and Human Services; ^10^Maryland Department of Health, Infectious Disease Epidemiology and Outbreak Response Bureau; ^11^Missouri Department of Health and Senior Services, Bureau of Communicable Disease Control and Prevention; ^12^Division of Epidemiology and Immunization, Massachusetts Department of Public Health, Bureau of Infectious Disease and Laboratory Sciences; ^13^Division of Epidemiology-Disease Surveillance and Investigation, District of Columbia Department of Health; ^14^Country Office, Dominican Republic, Division of Global HIV and TB, Center for Global Health, CDC.

Since 2013, CDC has received reports and investigated serious complications among medical tourists (i.e., persons whose primary purpose for international travel is medical care) upon their return to the United States (*1*). On May 1, 2017, the New York City Department of Health and Mental Hygiene informed CDC of three patients with nontuberculous mycobacteria (NTM) surgical site infections (SSI), all of whom had undergone cosmetic surgical procedures by a single surgeon at Centro Internacional de Cirugía Plástica Avanzada (CIPLA) in the Dominican Republic (*2*).

To identify additional patients, calls for cases were issued via CDC’s Epidemic Information Exchange (Epi-X), state-based health alert systems, the Infectious Diseases Society of America’s Emerging Infections Network, and the American Society of Plastic Surgeons’ email distribution list. State and local health department staff members interviewed reported patients to collect information about medical care received abroad, symptoms, and treatment received after their original surgical procedures. A confirmed case of cosmetic surgery–associated NTM infection was defined as a diagnosed SSI and laboratory evidence confirming the presence of NTM in a U.S. resident who underwent a cosmetic surgery procedure in the Dominican Republic since January 1, 2017.

As of November 8, 2017, CDC had been notified of 52 patients from nine states with an SSI after cosmetic surgery in the Dominican Republic; 38 (73%) met the confirmed case definition. The remaining 14 did not have laboratory evidence of NTM and thus did not meet the confirmed case definition. All confirmed cases occurred in women who reported undergoing surgery during January 4–July 14, 2017 (Figure). Patients meeting the confirmed case definition identified 14 surgeons at seven surgical centers in the Dominican Republic (clinics A, B, C, D, E, CIPLA, and one unknown clinic). Among confirmed cases with available information, 26 (81%) of 32 patients reported undergoing surgery at CIPLA; 11 of 11 with information on treatment received more than one antibiotic, and 14 of 15 required therapeutic surgical procedures after returning to the United States. One death was reported.

**FIGURE:**
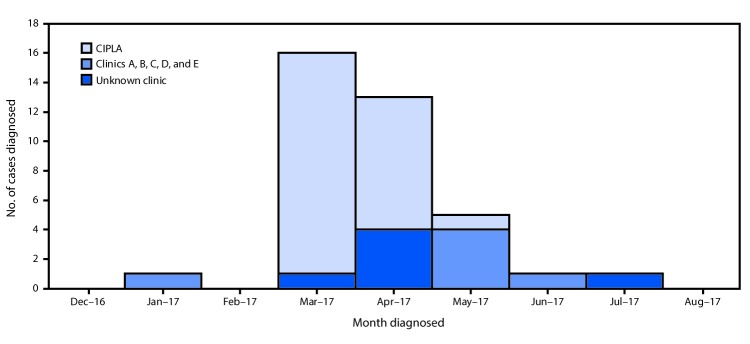
Nontuberculous mycobacteria infections (N = 37) associated with cosmetic surgery among U.S. medical tourists, by clinic and month of procedure — Dominican Republic, January–July 2017 **Abbreviation:** CIPLA = Centro Internacional de Cirugía Plástica Avanzada.

The New York State Department of Health Wadsworth Center conducted whole genome sequencing of isolates from 22 cases and identified three distinct genetic cluster variants. None of the clusters corresponded to a single clinic or a single surgeon. NTM are ubiquitous in nature and commonly colonize water systems as a mix of clonal variants, which can make speciation less relevant in the context of an outbreak.

CDC notified public health authorities in the Dominican Republic of the investigation and issued a travel notice on July 18, 2017, advising U.S. residents of the risks associated with any surgery at CIPLA (*2*). CIPLA was temporarily closed on July 8, 2017. 

Detection of outbreaks among medical tourists relies on clinical recognition and reporting to public health authorities. Patients who attend a single clinic abroad might be sparsely distributed across the United States. Furthermore, extrapulmonary NTM infections are not nationally notifiable and require targeted diagnostic testing, making cluster identification more difficult (*3*).

This investigation, in the context of medical tourism’s rapidly growing market, underscores the need for education of prospective medical tourists about possible risks and highlights the importance of health care providers having a high index of suspicion for NTM early in the evaluation of patients with SSI after cosmetic surgery (*4*). CDC continues to seek reports of infections after medical tourism from health departments. The Council of State and Territorial Epidemiologists recently approved a standard case definition to support improved surveillance for extrapulmonary NTM infections (*3*).
